# Association of Atypical Hemolytic Uremic Syndrome With Wilms' Tumor 1 Gene Mutations: A Case Series and Literature Review

**DOI:** 10.7759/cureus.70016

**Published:** 2024-09-23

**Authors:** Saeed Al Zabali, Sarah Alseneidi, Hassan Faqeehi, Sawsan Albatati, Abdulkarim Al Anazi

**Affiliations:** 1 Pediatric Nephrology, King Fahad Medical City, Riyadh, SAU

**Keywords:** atypical hemolytic uremic syndrome, denys–drash syndrome, global glomerulosclerosis, thrombotic microangiopathy, wt1 mutation

## Abstract

Atypical hemolytic uremic syndrome (aHUS) is a life‑threatening condition characterized by microangiopathic hemolytic anemia, thrombocytopenia, as well as acute kidney injury (AKI). It can occur primarily due to complement gene mutations or secondary to another underlying condition. Several cases with Wilms’ tumor gene 1 (WT1) mutations that presented with aHUS have been reported. Here, we report four cases of children diagnosed with WT1 mutations and presented initially with aHUS. There are two boys and two girls who presented with thrombotic microangiopathy (TMA), high lactate dehydrogenase, fragmented red blood cell (RBCs), and severe hypertension. All of them were anuric from the first presentation. Therapy with C5 inhibitors was initiated immediately and was associated with hematological remission without renal recovery. Renal replacement therapy (RRT) was started for all of the patients. A renal biopsy was conducted on two patients and showed global glomerulosclerosis. A genetic study identified pathogenic mutations in the WT1 gene. Two of the patients became dialysis dependent, and two patients underwent renal transplantation without the recurrence of aHUS. Our case series emphasizes that a diagnosis of WT1 mutation can be considered in children with aHUS with severe renal manifestations without a response to C5 inhibitors and with global glomerulosclerosis on renal biopsy. To our knowledge, this is the first report of a series of cases of WT1 mutations in pediatric patients presenting with clinical manifestation manifestations of aHUS. This unique finding highlights an association between HUS and WT1 mutation.

## Introduction

Atypical hemolytic uremic syndrome (aHUS) is an ultrarare disease caused by alternative complement pathway dysregulation [1,2]. Extrarenal manifestations can occur in up to 20% of patients [3]. Genetic or acquired dysregulation of the complement alternative pathway is detected in 40%-60% of patients with aHUS, suggesting a genetic predisposition [4]. Pathologic gene mutations result in loss-of -function in a complement gene (CFH, CFI, CD46, or THBD) or gain-of-function in an effector gene (CFB or C3) [3]. Noncomplement gene mutations like DGKE have been reported, and these cases are usually nonresponsive to the standard therapy [5]. Eculizumab is the first terminal complement blocker approved by the FDA for patients with aHUS [6]. Plasma therapy was the initial treatment for this condition, it was associated with death and chronic kidney disease in over 50% of patients, so the outcome for patients with aHUS has changed considerably following the introduction of eculizumab [7]. An in-depth analysis of the literature showed that nephrotic-range proteinuria is relatively common in patients with aHUS [8].

Denys-Drash syndrome (DDS) is caused by Wilms’ tumor gene 1 (WT1) gene mutations in exon 8 or 9, which encodes for a nuclear WT1 protein; this transcription factor plays an important role in the urogenital system development [9]. These WT1 variants have been confirmed to be pathogenic for DDS, which is characterized by nephrotic syndrome, abnormal kidney function, pseudohermaphroditism, and predisposition to Wilms' tumor [10]. The WT1 gene contains 10 coding exons at chromosome 11p13 [10]. It encodes a transcription factor that contains four Cys2-His2 ZF DNA-binding domains and a proline/glutamine-rich regulatory domain at the C-terminus and N-terminus, respectively [11]. It is an essential transcriptional and posttranscriptional regulator of gene expression. It acts as a master regulator of the mesenchymal-to-epithelial transition, which is necessary for nephrogenesis, although expressed in renal podocytes [1,12]. The prevalence of DDS is unknown. In the literature, fewer than 500 cases have been reported [13]. The management of WT1-related disorders includes three aspects: nephrectomy, renal transplantation, and focusing on disorders of sex development [9]. Several cases of WT1 gene mutation presenting with atypical hemolytic uremic syndrome (aHUS) have been reported [1,10,14]. 

Here, we report four cases of patients who presented initially with atypical hemolytic uremic syndrome (aHUS) and whose genetic analysis reports showed pathogenic mutations in WT1.

## Case presentation

Case 1

An 18-month-old boy, born full term with an unremarkable perinatal history, initially presented at five months of age to a local hospital with upper and lower gastrointestinal bleeding. Subsequently, he developed progressive edema, decreased urine output, and lethargy, followed by a seizure. On physical examination, he was lethargic and pale with respiratory distress, periorbital puffiness, and signs of fluid overload. He was hypertensive with a blood pressure reaching 140/110 millimeters of mercury (mmHg).The laboratory investigations during the first presentation are summarized in Table 1.

**Table 1:** Laboratory findings upon initial presentation LDH: Lactate dehydrogenase; ASO: anti-streptolysin O; ANA: anti-nuclear antibodies; ANCA: anti-neutrophil cytoplasm antibody;  anti-GBM: anti-glomerular basement membrane

Laboratory tests	Reference range	Case 1	Case 2	Case 3	Case 4
Hemoglobin g/dl	11.0-15.0	9	10	10	7
Platelets 10^9^/L	150000-450000	60000	56000	50000	75000
LDH U\L	125.00-220.00	946	648	792	929
Reticulocytes %	>0.5-<1.5 %	6.83	2.06	1.65	1.26
RBC fragments	Negative	Slight	Slight	Negative	Slight
Direct Coombs test	Negative	Negative	Negative	Negative	Negative
Creatinine umol/l	27.00-54.00	900	398	340	613
Urea mmol/L	2.50-6.00	39	42	50	42
Albumin g/L	38.00-54.00	27	24	22	26
C3 g/L	0.9-1.8	0.79	1.03	0.57	0.3
C4 g/L	0.1-0.4	0.18	0.3	0.1	0.1
CH50 U/mL	41.68-95.06	30.19	NA	40	53
ASO titer, ANA, ANCA, anti-GBM	Negative	Negative	Negative	Negative	Negative
Urine culture	Negative	Klebsiella pneumoniae	Negative	NA	Negative
Blood culture	Negative	Staphylococcal infection	Negative	Negative	Negative
Coombs test	Negative	Negative	Negative	Negative	Negative
ADAMTS-13 activity iu/ml	0.4-1.3	0.87	0.54	NA	0.9
24-hour urine protein g/day	<0.30 g/d	NA	2.68	NA	NA

The initial diagnosis was TMA, and a renal biopsy was performed; it was insufficient with four glomeruli and showed severe interstitial fibrosis, tubular atrophy, and glomerulosclerosis for all glomeruli. The patient's clinical status deteriorated despite intensive medical care, prompting the initiation of peritoneal dialysis (PD). He received the first induction dose of ravulizumab after receiving a meningococcal vaccination. He showed no improvement in renal and hematological parameters, and he had severe hypertension on five antihypertensive medications. Ten days later, plasmapheresis was begun for ten sessions with the re-induction of doses of ravulizumab. There was partial recovery of the hematological profile but no recovery of the renal parameters. His course was complicated by fungal peritonitis, which led to the removal of the PD catheter and the initiation of hemodialysis (HD). Unexpectedly, the gene study resulted in the diagnosis of autosomal dominant WT1-related disorder c.1405G>A p.(Asp469Asn). His phenotype is male, with unilateral undescended testis and absent right testis. The karyotype confirmed XY chromosomes. Despite ongoing supportive care, complement inhibitors, and plasma exchange, the patient remained anuric and hypertensive, necessitating chronic dialysis.

Case 2

A 23-month-old girl, who was previously healthy, developed nonbloody diarrhea, edema, and lethargy. Her family sought medical advice in a private clinic, and her initial laboratory tests showed AKI; she was transferred to our center. Upon admission, she had generalized edema and severe hypertension (systolic blood pressure 190/154 mmHg). She had anemia and thrombocytopenia as well as stage-3 AKI (Table 1). She was admitted to the pediatric intensive care unit (PICU) with an impression of thrombotic microangiopathies (TMA). Urgent continuous kidney replacement therapy (CKRT) was initiated. She received her first loading dose of ravulizumab, but there was no improvement in her renal function. Her hematological profile continued to deteriorate, necessitating multiple blood transfusions. A renal biopsy revealed advanced chronic thrombotic microangiopathy. Moreover, four of six glomeruli were globally sclerosed with severe interstitial fibrosis and tubular atrophy. She received a second and third dose of ravulizumab but remained anuric and hypertensive. Hemodialysis was continued at a frequency of five sessions per week. Genetic testing revealed CFHR1/CFHR4 gene deletion with a risk allele of uncertain significance. Further whole-genome analysis was consistent with autosomal dominant WT1, NM_024426.5:c.1399C>T. A segregation analysis was performed for both parents, with the father showing wild type and the mother being a heterozygous carrier of the deletion encompassing the CFHR1 and CFHR4 genes. The patient underwent a renal transplantation and is now three years old. She has completed six months posttransplant without any signs of recurrent aHUS.

Case 3

**Figure 1:**
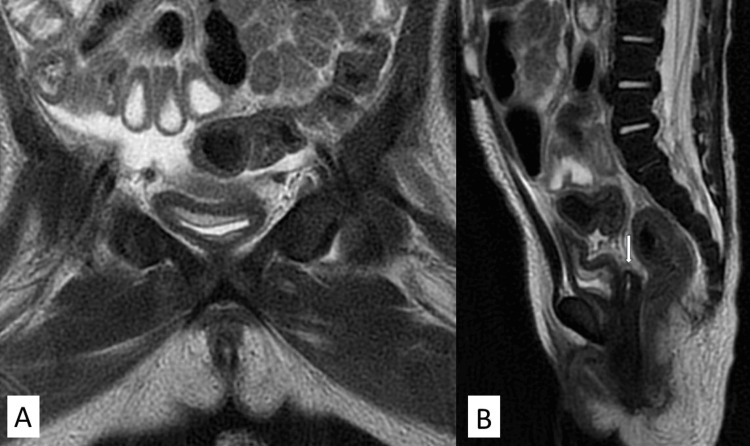
MRI. A: Coronal TWI demonstrates no ovaries; B: sagittal T2: Small blind-ended  tubular structure noted posterior to the urethra and anterior to the rectum most likely representing the vestigial vagina MRI : Magnetic resonance imaging; TWI: track weighted imaging

A six-month-old girl presented with abnormal movements, edema, and AKI. On examination, the patient was hypertensive and fluid overloaded. Laboratory investigations revealed AKI ,anemia, and thrombocytopenia (Table 1). She was admitted to the PICU for oliguria and generalized edema and required intubation. Subsequently, she required a labetalol infusion for a hypertensive emergency. CRKT was begun. The patient still presented with anemia and thrombocytopenia, with anuria that was not responding to medical management. There was difficulty controlling her blood pressure with the use of three antihypertensive medications. The first dose of eculizumab started without any clinical improvement in her urine output, but there was normalization of the platelet levels and then hemoglobin levels. The second and third doses of eculizumab were received without improvement in her renal parameters. A PD catheter was inserted. She was discharged home and then readmitted after one month with refractory peritonitis and septic shock. An abdominal ultrasound and pelvic MRI revealed the absence of internal female reproductive organs (Figure 1).

The absence of ovaries was further confirmed by laparoscopy, which was performed after reinserting the PD catheter. This patient was morphologically female with normally female external genitalia but confirmed to be a genetic male because of karyotype 46: XY. Her aHUS panel came back negative, but the wall echo shadow (WES) showed a heterozygous pathogenic variant in the WT1 gene (c.1405G>A, p.Asp469Asn, NM_024426.6) and heterozygous VOUS in the ANLN gene (9c.1868A>G, p.Asp623Gly, NM_001284301.3). The child’s diagnosis fits with autosomal dominant DDS. The patient underwent renal transplantation and is now five years old. She has been four months posttransplant without any signs of recurrent of aHUS.

Case 4

A one-year-old boy, who was previously healthy, had a hypospadias repair two weeks before his presentation. He arrived with a history of vomiting, watery stools, generalized edema, and decreased urine output for five days. In the emergency department, he developed an abnormal movement for two minutes. The initial investigations showed low hemoglobin and platelets, elevated lactate dehydrogenase (LDH) and schistocytes in the peripheral smear (Table 1). He was admitted to the PICU for respiratory support. CKRT was commenced. He exhibited a persistent drop in his hematological parameters with signs of hemolysis, so 10 sessions of plasmapheresis were undertaken without improvement in his hematological and renal parameters. Eculizumab treatment was started. His hemoglobin and platelets were normalized. The genetic study results were heterozygous for WT1 g.32414283C>T. However, the patient did not recover renal function, and he became dialysis dependent.

## Discussion

The WT1 gene encodes a transcription factor that contains four Cys2-His2 ZF DNA-binding domains and a proline/glutamine-rich regulatory domain at the C-terminus and the N-terminus, respectively [11]. WT1 was expressed in developing podocyte cells, but this expression decreased as the renal corpuscle matured [15]. It encoded proteins that are essential for regulating cell growth and maintaining podocytes' function [16]. With the development of genetic tests, it is now recognized that WT1-related disorders involve a broad phenotypic spectrum. This insight emphasizes the importance of thorough genetic testing and management strategies for patients suspected of having WT1-related disorders [9]. The constitutional variants in the zinc finger (ZF) motif of the WT1 gene have been established to be associated with DDS [17].

We report four cases of patients who presented with hemolytic anemia, thrombocytopenia, and AKI. All of the patients presented with a severe course of significant edema and decreased urine output, as well as hypertension. Additionally, two of them presented with nonbloody diarrhea (patients 2 and 4), one patient had gastrointestinal bleeding (patient 1), and another developed abnormal movement (patient 3). Five similar cases have previously been reported (Table 2).

**Table 2:** The clinical findings of the reported cases as well as our cases with WT1 gene mutations and aHUS presentation CFHR1,4: Complement factor H-related protein 1,4; PD: peritoneal dialysis; ESRD: end-stage renal disease; GI: gastrointestinal; aHUS: atypical hemolytic uremic syndrome; WTI: Wilms’ tumor gene 1; TMA: thrombotic microangiopathy; MPGN: Membranoproliferative glomerulonephritis; DMS: diffuse mesangial sclerosis; SNPs: single nucleotide polymorphisms

	Current study				Case 5	Case 6	Case 7	Case 8	Case 9
Cases	Case 1	Case 2	Case 3	Case 4	Sherbotie et al. [14]	Sherbotie et al. [14]	Alge et al. [1]	Manivel et al. [18]	Cheng Cheng [10]
Age of presentation (months)	5	23	6	12	13	16	8	24	24
Karyotype	46, XY	46, XX	46, XY	46, XX	46, XY	46, XY	46, XX	46, XY	46, XY
Morphological sex and genital abnormality	Male, unilateral undescended testis, and absent right testes	Female	Female, absent internal female reproductive organs	Male, hypospadia	Male, with grade III hypospadias and undescended testes	Male with grade III hypospadias with bifid scrotum and undescended testes	Female with absent ovaries	Male, ambiguous external genitalia, bilateral Mullerian, and right Wolffian	Female external genitalia
Initial signs and symptoms	GI bleeding	Nonbloody diarrhea, progressive generalized edema, and lethargy	Abnormal movements with edema	Vomiting and non-bloody diarrhea, oliguria, and edema	Progressive edema, anorexia, and irritability	Irritability, vomiting, edema, and decreased urine output	Oliguria, hypertension, vomiting	HUS	Rashes, abdominal pain, nonbloody loose stools, edema, anuria
Hypertension	Yes	Yes	Yes	Yes	No	Yes	Yes	Yes	Yes
Nephrotic range proteinuria	Not available Patient was anuric	Yes	Yes	Yes	Yes, a month before presentation	Yes, at presentation	No	-	No
C3 level	Normal	Normal	Decreased	Decreased	Normal	Normal	Decreased	-	Normal
Renal biopsy findings	Severe interstitial fibrosis and tubular atrophy	4 of 6 glomeruli globally sclerosed with severe interstitial fibrosis and tubular atrophy ,chronic thrombotic microangiopathy	Not conducted	Not conducted	Microangiopathic glomerulopathy with ESRD findings	Microangiopathic glomerulopathy with ESRD findings	3 of 8 glomeruli were globally sclerotic and active TMA	Initial biopsy: MPGN Gross specimens: DMS and metanephric hamartoma	Gross specimens: DMS and glomerular dysplasia
Treatment	Ravulizumab and plasmapheresis	Ravulizumab	Eculizumab	Eculizumab	Plasmapheresis	-	Plasma infusions/exchange, eculizumab	-	Plasma exchange
Gene	WT1	WT1	WT1	WT1	WT1	WT1	WT1	-	WT1
Variant	NM_024426.4:c.1405G>A	NM_024426.5:c.1399C>T	c.1405G>A chr11:32413560 p.Asp469Asn g.32413560C>T	c.1268 G>A (p.Cys 423 Tyr)	IVS9+111 C>T	p. Arg394Trp in exon 9	p. Arg394Trp in exon 9	-	p. Asp252Asn in exon 9
Classification	Pathogenic	Pathogenic	Pathogenic	Pathogenic	Variant not in clinvar	Pathogenic	Pathogenic	-	Pathogenic
Zygosity	Heterozygous	Heterozygous	Heterozygous	Heterozygous	Heterozygous	Heterozygous	Heterozygous	-	Heterozygous
Complement abnormality	Not found	CFHR1 (NM_002113.2; NGS-CNV analysis + MLPA) CFHR4 (NM_001201550.3; NGS-CNV analysis + MLPA). Classified as risk allele of uncertain significance. Heterozygous deletion encompassing the entire CFHR1 and CFHR4 genes	Not found	Not found	-	-	Autoantibody (-) CFH H3 haplotype with three SNPs	-	Not found
Outcomes	Dialysis	Dialysis, renal Transplantation. No recurrences of HUS for 7 months	Dialysis, renal transplantation. No recurrences of HUS for 5 months	Dialysis	Renal transplantation. No recurrences of HUS for 4.5 years	Renal transplantation. No recurrences of HUS for 11 months	PD; bilateral nephrectomy at 13 months old	Renal transplantation at 32 months old	Renal transplantation. No recurrence of HUS

Similarly, all of these patients presented with edema, decreased urine output, and hypertension. Manivel et al. described a 26-month-old male who presented with HUS. The karyotype confirmed XY chromosomes. He was born with ambiguous genitalia [18]. Sherbotie et al. reported two patients with DDS who also had the presentation of aHUS [14]. The first patient was a 16-month-old boy who developed aHUS and DDS at the same time. The second was a 13-month-old boy who developed aHUS 1 month after nephrotic syndrome and a urinary tract infection. Alge et al. reported an eight-month-old female infant who had aHUS with the first presentation of aHUS [1]. Cheng Cheng et al. recently reported a case of a two-year-old child who was diagnosed with DDS as the initial presentation of DDS [10]. Two cases have been reported in adult patients, both of them presenting with clinical systemic lupus erythematosus (SLE) and aHUS; their genetic testing was positive for WT1 mutations [19].​​​​​​ 

Two of our patients had C3 levels indicating complement activation, as in the case reported by Alge et al. In contrast to the previously reported cases, except for the case reported by Alge et al., all of our patients were treated with C5 inhibitors. Patient 1 received plasmapheresis, as in the case reported by Sherbotie et al., Alge et al., and Cheng Cheng et al. Renal biopsies were performed on two of our patients, showing severe global sclerosis with severe interstitial fibrosis and tubular atrophy. Among the previous cases, two underwent renal biopsies at the time of the aHUS diagnosis, which showed evidence of TMA. Later, diffuse mesangial sclerosis (DMS) was proven by means of bilateral nephrectomy when transplantation was performed, indicating that aHUS occurred before DMS developed. DMS was also detected in three other cases a few years after onset of the disease. Despite all of the treatments, all of our patients progressed rapidly to ESRD and required RRT. Therefore, these four cases prove an apparent association between aHUS and WT1 mutations.

Regarding podocytopathy, the findings of proteinuria at presentation indicate underlying podocyte injury. These cases add to a growing literature linking aHUS with podocyte dysfunction and glomerulopathy. Noris et al. have mentioned that the loss of podocyte-derived vascular endothelial growth factor (VEGF) contributes to the dysfunction of glomerular endothelium which in turn leads to the development of aHUS. We hypothesize that our patient's WT1 mutations resulted in a podocytopathy that led to endothelial dysfunction, which, combined in one patient with CFHR1,4, culminated in the development of aHUS. All of our patients have a heterozygous pathogenic mutation of WT1. Patient 2 has an additional mutation of CFHR1/CFHR4. Initially classified as likely pathogenic and then reclassified to a risk allele of uncertain significance, this mutation is associated with aHUS. In contrast, in the case reported by Alge et al., nonpathogenic single nucleotide polymorphisms (SNPs) in the CFH gene are associated with the development of aHUS. Patient 3 has an additional heterozygous mutation classified as VOUS in the ANLN gene, which encodes an actin-binding protein that plays a role in cell growth and migration and in cytokinesis.

Although the mechanisms remain unclear, studies suggested that reduced WT1 expression, overexpression of transforming growth factor-β1 (TGF-β1), upregulated Pax-2 expression, and platelet-derived growth factor-α (PDGF-α) play important roles in this condition [20]. Interestingly, VEGF is important for the survival of endothelial cells, podocytes, and mesangial cells, and it is secreted by podocytes. VEGF's decreased availability can lead to glomerular endothelial dysfunction in nephrotic syndrome [8]. Therefore, abnormal podocytes disturb the glomerular filtration barrier integrity, which results in significant proteinuria in DDS, while nephrotic-range proteinuria enhances the release of different procoagulants that make those patients with a high risk of TMA ​​​​[8].

## Conclusions

A two-way association occurs between podocyte dysfunction and aHUS pathophysiology. On the one hand, nephrotic-range proteinuria might happen in patients with aHUS. On the other hand, podocyte dysfunction can lead to nephrotic-range proteinuria, which puts the patient at risk of developing thrombotic microangiopathy by inducing prothrombotic abnormalities and endothelial dysfunction. To our knowledge, this is the first report of a series of cases of WT1 mutations in pediatric patients presenting with clinical manifestations of aHUS. Further functional studies of WT1 mutations are needed to elucidate the association between WT1 and aHUS and to develop possible treatments. 
